# Probably Correct: Rescuing Repeats with Short and Long Reads

**DOI:** 10.3390/genes12010048

**Published:** 2020-12-31

**Authors:** Monika Cechova

**Affiliations:** Genetics and Reproductive Biotechnologies, Veterinary Research Institute, Central European Institute of Technology (CEITEC), 621 00 Brno, Czech Republic; cechova.biomonika@gmail.com

**Keywords:** repeats, satellite, multi-mapping, reference, long reads

## Abstract

Ever since the introduction of high-throughput sequencing following the human genome project, assembling short reads into a reference of sufficient quality posed a significant problem as a large portion of the human genome—estimated 50–69%—is repetitive. As a result, a sizable proportion of sequencing reads is multi-mapping, i.e., without a unique placement in the genome. The two key parameters for whether or not a read is multi-mapping are the read length and genome complexity. Long reads are now able to span difficult, heterochromatic regions, including full centromeres, and characterize chromosomes from “telomere to telomere”. Moreover, identical reads or repeat arrays can be differentiated based on their epigenetic marks, such as methylation patterns, aiding in the assembly process. This is despite the fact that long reads still contain a modest percentage of sequencing errors, disorienting the aligners and assemblers both in accuracy and speed. Here, I review the proposed and implemented solutions to the repeat resolution and the multi-mapping read problem, as well as the downstream consequences of reference choice, repeat masking, and proper representation of sex chromosomes. I also consider the forthcoming challenges and solutions with regards to long reads, where we expect the shift from the problem of repeat localization within a single individual to the problem of repeat positioning within pangenomes.

## 1. Introduction

While next-generation sequencing is increasingly used both in research and clinical practice, a subset of sequencing reads is frequently underutilized. These are reads that cannot be uniquely positioned within their respective genomes, and are thus multi-mapping in the chosen reference assembly. They frequently originate from duplicated genes [1], transposable elements [2,3], satellite repeats (e.g., centromeric and telomeric reads) [4], and more generally a heterochromatic portion of the genome [5]. Indeed, an estimated 50–69% of the human genome is repetitive [6,7], as is as much as 80% of the maize genome [8]. Multi-mapping reads are also an unavoidable consequence of segmental and whole-genome duplications [9,10]. Additional sources of nearly identical sequences are allelic variants and haplotypes.

The two most important parameters for whether a read is multi-mapping are the read length and genome complexity, whereas genome complexity can also be defined in terms of the length of repetitive units, i.e., length-sensitive [11]. Importantly, in order to characterize the repeat array, the reads need to be longer than *r*, where *r* is the length of the repeat unit. What is the minimal sequencing read length required to capture the repeat array? During PCR design, two ~20bp oligonucleotides can be anchored in the genome. Yet, such a reaction could still yield an unspecified product if one or both primers were positioned in the repetitive regions, requiring an optimization of the PCR reaction. Thus, the repetitiveness of any genome can be defined in terms of read length required to successfully assemble it. The k-mer uniqueness ratio is defined as the percentage of the genome that is covered by unique sequences of length *k* or longer [12]. For a variety of organisms, a *k* of at least 50 is required to cover a significant portion of their respective genomes [12]. The mappability, where the genome is divided into windows of size *k*, and the uniqueness of each window is calculated (i.e., mappability of 0.5 means exactly two identical windows exist in the genome), can be calculated for any *k* (e.g., with the GEM library toolkit), and provided as a genome track. Note that for human, as much as 28.4% of reads are unmappable to the assembled portion of the human genome with the read length 20, while only 2% with the read length 200 [13,14]. These numbers refer to the assembled portion of the human genome, which increased significantly from hg19 to hg38. The assembled portion impacts called variants [15], and is expected to rise further with the assembly by the Telomere-to-Telomere (T2T) consortium. 

Along with the technical variation (read length/error rate), there is a substantial biological variability outside of the reference genome sequences, especially in the form of satellite repeats. According to the “satellite library” hypothesis, an initial set of sequences can lead to the vast variability of outcomes, generating changes in sequence and copy number in individuals and populations [16]. Indeed, among human populations, the centromeric array of the X chromosome can vary by an order of magnitude (0.5–5 Mb) [17]. Wei and colleagues showed that repeat clustering did not recapitulate the expected relationships in geographically separated populations of *Drosophila* [18]. In great apes, Cechova and colleagues described vast variability among satellite repeats in great apes [19]. Intriguingly, different sequencing technologies provide different repeat estimates, although they agree qualitatively (abundant versus rare repeats) [19]; I discuss some potential reasons later. Approaches and software for satellite biology are reviewed in [20] and include both short- and long-read solutions.

Because of the intrinsic difficulty of dealing with repetitive parts of the genome, sometimes it might be advantageous to remove the repetitiveness in order to study underlying biological processes, such as cell division. As an example, an artificial, non-repetitive centromeric region was created to study centromere genomics with the use of human artificial chromosomes (HACs) [21]. Last, next-generation sequencing reads, even if not multi-mapping, can fall short of capturing the full repeat variability of individuals [22], especially when compared to a single reference genome (i.e., limited representation of a genome).

## 2. Reference Genomes Are Inherently Incomplete 

Reference genomes represent a simplified, linear representation of the conceivable version of a genome of a given species [23]. Such references are incomplete: even the best representations contain gaps in difficult, heterochromatic parts of the genome [24]. Moreover, as much as 5–10% of the human genome remains poorly characterized [25], and up to tens of percentage points might be completely missing in other organisms, such as birds [26], all while underestimating the copy number of repetitive regions. This is because high-identity regions are often collapsed during the assembly process from short sequencing reads [27] or long erroneous reads. As an example, only <0.1% of GRCh38 reference is composed of repeats HSAT2/3 but as much as 2.6% of read bases are HSAT2/3 [28]. When dealing with such hard-to-assemble regions, it might be advantageous to use “the most likely representation”, rather than the reference assembled from any living individual [17]. This idea has been implemented for centromeric arrays [17]. Instead of a multi-megabase gap as in previous human reference genomes, GRCh38 centromeres are composed of these presumed sequences, based on the second-order Markov models of monomer variants. Still, complete assembly is the ultimate solution to repeats [29].

Recently, a newly established Telomere-to-Telomere consortium aimed to assemble human chromosomes in full and present a new (near) complete sequence of a human genome [30,31], including the first complete sequence of the human chromosome X [32]. However, gaps in reference genomes are not the sole reason why reference assemblies are generally incomplete. The variability among individuals of a given species means that the full sequence content simply cannot be captured by looking at a single individual [33,34,35,36]. A recent study identified an additional 300 Mbp of sequences (although predominantly HSAT2 and HSAT3) that were not represented in the human reference (GRCh38) but found among 910 individuals of African descent [35]. Another study found 46 Mb in 1000 Swedish individuals [36], complementing a previous study that performed de novo assemblies of two Swedish genomes and revealed as much as 10 Mbps of novel sequence (originating from centromeric and telomeric regions and the chromosome Y), almost one-third of which was different from any sequences present in existing nucleotide databases [37]. The degree to which an individual is represented by a reference also depends on ancestry. The individuals that do not match the ancestry of the particular reference genome built might be misrepresented, leading to false variant calls or uninterpretable GWAS results [38,39]. One of the options is to use ancestry-specific reference builts—an example of which might be the Japanese reference genome [40]. Similar considerations apply to different haplogroups, but, as expected, representing all haplotypes with a contig each leads to a multi-mapping problem (contig from a specific haplotype is referred to as a haplotig). In summary, reference genomes are either incomplete or introduce multi-mapping issues at the allelic, haplogroup, or chromosomal level.

The downstream analysis—such as mapping accuracy, gene expression analysis, and calling of structural variants—are affected by the following: (1) the specific reference genome (that comes in multiple private and public versions) [41], (2) whether or not the genome is repeat masked, and (3) the representation of pseudoautosomal regions (PARs) and alternative haplotypes. The understanding of the reference genome as the representative species genome should be uncoupled from the sequence that is to serve as an alignment reference [23]. First, for typical applications, it might be advantageous not to use alternative haplotigs and to mask large multi-copy sequences such as PARs and a small subset of α-satellites that are artificially identical in the current GRC reference genomes [41]. In this scenario, one must wary that variant calls from these regions might originate from more than one genomic region. However, not including unplaced and unlocalized contigs might force reads from these contigs to be mapped to the chromosomal part of the reference and again lead to false variant calls. On the other hand, aligners typically assign mapping quality 0 to multi-mapping reads, and such reads might be ignored by downstream pipelines.

Sometimes, multiple reference genomes are concatenated and used as a mapping target: good examples are the inclusion (or a lack thereof) of a mitochondrial genome or sequences of spike-in controls. Crucially, even if one is interested only in a single chromosome, sequencing reads still need to be mapped to a full reference genome. This is because if no other chromosomes are offered as a mapping target, the read counts will become overrepresented—as much as one-third of all sequencing reads could map to a single chromosome in the case of the human genome due to repeats; this proportion drops significantly when repeat masking is in place (see Table 1). Second, genes can have repetitive parts (in both exons [42] and introns [43,44]) and intergenic regions can be low-complexity; thus, repeat masking the reference genome will result in an increase in the proportion of unmapped reads (Table 1). Third, some parts of the genomes, e.g., repetitive heterogametic sex chromosomes (chromosome Y in mammals and chromosome W in birds), are often underrepresented. In summary, the particular version of the reference genome must be carefully considered and chosen contingent on the desired application.

## 3. Short Reads 

**Figure 1:**
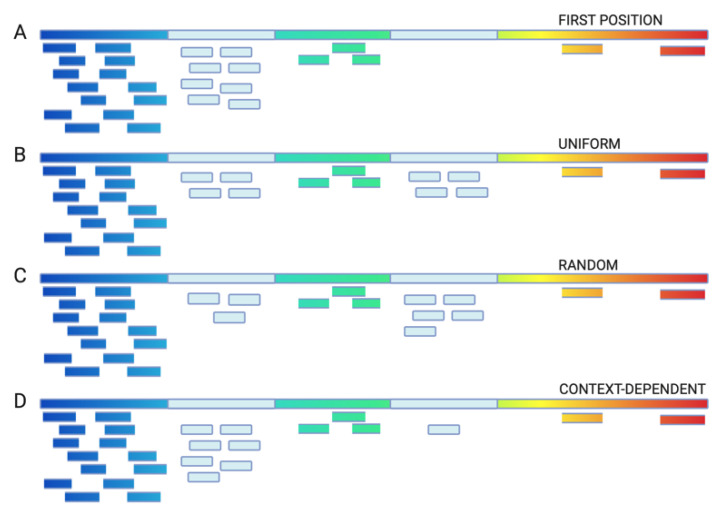
The mapping strategies for the multi-mapping reads. Reference genome (rainbow) with two duplicate regions (light blue). The multi-mapping reads (light blue) are distributed among mappings based on (**A**) the first position or the grouped assignment, (**B**) uniformly among all equally good mappings, (**C**) randomly, or (**D**) context-dependent assignment based on the coverage of neighboring sites. Figure created with BioRender.com.

Repetitive regions are hard to resolve and are variable among individuals and technologies: both biological and technical variability is present. If the reads are mapped to such repetitive reference, how should the multi-mapping reads be dealt with? Four main approaches exist: use first position or the “grouped assignment”, uniform assignment, random assignment, and, last, context-dependent distribution (Figure 1).

### 3.1. Methods of Multi-Mapping Read Assignment

The first approach maps reads to the first possible position out of multiple equally good options (Figure 1A). If no further post-processing is applied, this approach can lead to erroneous downstream conclusions (e.g., in early versions of Tophat all multi-mapping reads were in some instances aligned to the same locus). For example, if for all duplicated genes, only the first annotated gene copy is used as a mapping target, which then biases read counts and an expression value towards the first gene/isoform. Alternatively, all such reads could be annotated as a group: presenting both unique regions and the expression of the “unassigned, multi-mapping group”.

The second approach (although rather theoretical) assigns all multi-mapping reads uniformly: the same number of reads is assigned to each of the mapping targets (Figure 1B). While this might not reflect the biological reality, at least it does not favor any particular instance of a repeat.

The third approach represents a naive allocation of multi-mapping reads that randomly separates reads into one of the “equally good” positions (Figure 1C). This is typically a default option in many short-read aligners, including bwa [45], bowtie [46], HiSAT [47], and STAR [48]. In this implementation, multiple runs will yield slightly different results.

The fourth approach represents one of the more sophisticated approaches that aim to gauge which option is more likely biologically. 

Last, reference-free approaches can be applied, such as read clustering. For example, the authors of [49] attempted to characterize satellites directly from short unassembled reads, using clustering and visualizations for which they offered biological interpretations [49].

The choice of the multi-mapping strategy can artificially bias read counts related to specific annotations, yet I believe that the effect sizes of these strategies are largely unknown in the scientific literature.

### 3.2. Multi-Mapping Reads in RNA-Seq, Chip-Seq, Hi-C, and Exome Sequencing

For RNA-Seq, the most common pipelines deal with multi-mapping reads as follows; HTSeq-count and STAR geneCounts ignore them, while Cufflinks can either split reads equally or use uniquely mapped reads as guidance; the latter option could be problematic for small noncoding RNAs, especially those located in the introns [50]. For a comprehensive review on handling multi-mapping reads in RNASeq datasets, see the work in [50]. In general, it is believed that the expression levels for the genes that contain multi-mapping reads are underestimated [51]; and that hundreds of genes, many of which are relevant for human health, could be affected [51]. One solution is to use group-level expression (for a set of genes) to circumvent the multi-mapping problem [51]. Thus, read counts could be calculated separately for the input genes and merged genes, as was implemented in the tool mmquant, applying a gene clustering strategy [52]. More sophisticated approaches involve hierarchical allocation of reads: first resolving ambiguities among genes, followed by isoforms and individual alleles. A similar approach was implemented in the software MMSEQ [53] or EMASE (Expectation-Maximization for Allele-Specific Expression) [54]. Algorithms implementing EM or Expectation-Maximization are important for assigning multi-mapping reads in RSEM [55], and for pseudoalignments with Kallisto/Salmon [56,57]. Instead of mapping reads to a reference, pseudoalignments estimate which transcripts could have generated them. As short sequencing reads still contain a limited number of sequencing errors, some methods have attempted to extend the array of possible mapping contexts, thus accounting for these errors, in order to identify the most likely mapping in RNA-Seq experiments [58].

Analogous approaches have been used for ChIP-seq datasets; when analyzing transcription binding sites, some regions in the genome are known to bind transcription factors based on the information from the uniquely mapped reads. Thus, one might choose to distribute multi-mapping reads preferentially to those regions (Figure 1D). Zhang and colleagues have proposed models that use local concentrations of directional reads and account for local genome repetitiveness (using whole-genome read mappability profiles) while differentiating between adjacent binding sites [59].

The typical pipelines for a chromosome conformation capture, such as Hi-C, filter out multi-mapping reads, as they are not considered useful for delineating chromatin interactions. However, this disproportionally affects repetitive parts of the genome, such as the Y chromosome, where ampliconic regions span as much as almost half and over half of the male-specific euchromatic proportion in human and chimpanzee, respectively [60]. Ampliconic regions host large inverted repeats, i.e., palindromes, which are then omitted from traditional pipelines. Using a probabilistic approach implemented in the package mHi-C [61], Cechova, Vegesna, and colleagues were able to analyze palindromic arms of human palindromes and found a higher density of chromatin interactions; none of these regions could be analyzed if multi-mapping reads had been excluded [62].

Last, multi-mapping reads also affect whole-genome and exome sequencing data, as well as small RNAs [63], where the effect is especially pronounced due to biologically constrained read lengths, and finally metagenomics, where only subtle differences might exist between various strains [64].

### 3.3. Repeat Masking and Its Consequences

Repeat masking refers to a process in which the underlying sequence gets marked as repetitive (typically with repetitive parts in lowercase letters: soft-masking) or fully suppressed (typically replaced by Ns/Xs: hard-masking). Soft-masking is relevant whenever the specific part of the sequence is visually inspected (e.g., during primer design or when examining gaps in the assembly), as a repeat-masked sequence might hint why the given region was challenging to analyze. In contrast, hard-masking might be advantageous in specific applications (e.g., the pseudoautosomal region on the chromosome Y is typically hard-masked). Importantly, masking will not only affect heterochromatic parts of the human reference genome but also genes and other regulatory sequences that may carry repeats. In summary, repeat masking (especially hard-masking) will have an effect on all downstream processes that depend on the read mapping, including, but not limited to, variant calling, gene expression, and chromatin capture analysis.

### 3.4. Sex Chromosomes

In order to build a new reference genome, only the homogametic sex is typically sequenced, as heterogametic sex chromosomes are harder to assemble at any given sequencing coverage (there is always fewer data for a given sex chromosome compared to an autosome) and because chromosomes Y/W tend to be repetitive [65]. The omission of sex chromosomes is especially prominent in the GWAS studies [66,67,68]. Sex chromosomes require special considerations during the mapping process. The reads from chromosomally male and female individuals will align differently depending on whether the reference even contains the Y/W chromosome [69]. The tool XYalign can identify XX and XY individuals across different experimental conditions (including low-coverage samples and exome sequencing), as well as improve variant calling on sex chromosomes [69]. Moreover, X and Y chromosomes share a pseudoautosomal region, in which homologous sequences have a high degree of identity and still recombine. Accounting for sex chromosomes can increase the number of unique genes identified as differentially expressed between the sexes and to increase expression estimates in the pseudoautosomal region of the X chromosome [70].

## 4. Long Reads 

The biggest promise of long (yet erroneous but see HiFi reads) reads is to span difficult, repetitive, and heterochromatic regions in the genomes or populations of interest [71,72,73,74]. Long reads, especially in combination with other orthogonal technologies enhancing the assembly contiguity (such as chromatin captures/Hi-C [75] or optical maps [76]), are now turning near-complete or complete reference genomes into reality [31,32,77]. Additionally, traditionally difficult regions, such as the Major Histocompatibility Complex (MHC), have been recently characterized in detail with PacBio and Nanopore sequencing [73,78,79,80,81]. This opens up the possibility to also survey the satellite content, either directly from long reads [82] or from assemblies/references [83,84]. Hundreds of kilobase pairs in read length ensure that many repeat arrays are fully encompassed within the sequencing reads. Indeed, Cechova and colleagues demonstrated that depending on the species, 90–95% and 99% of abundant repeat arrays were fully nested within individual reads in Nanopore and PacBio, respectively [19]. Moreover, the intra-repeat array variability was present: among the 39 most abundant repeats in the great ape genomes, at least 10–25% of all arrays were composed of a mix of different repeated motifs [19]. Such satellites can either be surveyed de novo (when the set of repeats in the genome is unknown; this is difficult due to the intermixing of sequencing errors and rare variants) or by searching for a specific set of repeats when the presumed errors can be “canceled out”, as was implemented in the Noise-cancelling repeat finder (NCRF) [82]. Specifically, NCRF can identify long satellite arrays in Nanopore and PacBio reads, notwithstanding their length or error rate. However, the variability in such satellite arrays is challenging to capture in standard file formats such as Variant Call Format (VCF). Comparing VCF files across experiments requires accounting for both the combination of sequencing errors (potentially shifting starting positions) and tandem representation of biologically imperfect repeats. Even in the most simple scenario of small Illumina variants, multiple equivalent representations are possible [85]. The tools for the reconciliation of long tandem arrays, and their comparisons, are currently being developed [86]. Satellite arrays inside the existing assemblies/references can be curated, e.g., with TandemTools, a novel tool for the polishing and quality assessment of extra-long tandem repeats (ETRs) [87]. Specific long-read mappers can be used to align reads to highly repetitive reference sequences [88], while accounting for the allele bias (so that non-reference allele within a repeat does not penalize the alignment) [89]. In summary, long reads are much better equipped to characterize the variable satellite content and to assemble and span difficult, repetitive parts of the genome.

### 4.1. Long-Read Sequencing Strategies

The biggest challenge before fully utilizing long reads is their elevated raw error rate, previously (2019) reported at 14.90% and 16.10% in PacBio and Nanopore, respectively, and continuously improving since. The most recent reports suggest 95% (and higher) accuracy for raw Nanopore reads [90], especially due to the improvements in pore design and basecalling algorithms. Raw reads can further be used to build consensus, which is the preferred strategy for PacBio; consensus HiFi reads are both long (>10 kbp), and accurate (>99.9%) [91,92]. This is because HiFi reads require circularizing of the DNA, so that it can be read multiple times over to increase accuracy, rendering them an effective technology for the genome assembly problem (see below).

PacBio and Nanopore differ in their estimates of the repeat copy number and overall repeat content, even for the same individual [19,83] and also present with a strand bias [83]. The repeat content differs even after sequencing reads are subsampled to the common length distribution for both technologies, to account for the potential differences in read lengths [19]. The potential explanations include distinct library preparation protocols, DNA damage, DNA quality (that could potentially decline during prolonged sequencing at room temperature), and non-canonical DNA structures, as reviewed in [19]. Thus, some of the repeats discovered using just one technology might not be confirmed in another, and vice versa.

The multi-mapping problem I described for the short reads has in some ways shifted to larger scales—from arrays spanning a few kilobases we are now able to resolve megabase-long arrays. However, the sequencing errors still disorient the aligners both in accuracy and speed [93]. The alignments are bound to contain some “chance” alignments due to a matching subset of “chance” nucleotides. With the continued progress in basecalling, it is expected that the error rates will continue to decline, and that additional factors (such as epigenetic modifications) will be responsible for the ambiguous calls.

### 4.2. Differentiating (Nearly) Identical Repeat Arrays

Small variants in otherwise homogeneous repeat arrays are especially useful to aid in the assembly process and can be used to create a tiling path across repeats. For example, these distinct unique markers were reported on average every 2.3 kb in the centromeric satellite array on the X chromosome (DXZ1), with a maximum spacing of 42 kb [32]. Such spacing makes long reads especially useful in delineating repetitive arrays based on their unique markers, and assemblers HiCanu [91] and hifiasm [29] capitalize on this property. However, what if no unique markers are available? Is there a way one could differentiate between identical sequences of the same origin? What if we could, together with the sequence information, also capture the epigenetic modifications associated with these sequencing, and use them to differentiate among otherwise identical sequences to provide an “epigenetic phasing”? This is now possible with both PacBio and Nanopore, for PacBio using kinetic profiles (and recording the pausing of the polymerase) [94] and for Nanopore with electric signals (methylated bases modulate the raw signal) [95]. This can be done either directly [96,97,98] or through a base conversion in which a matched modified sample is created [99,100]. Therefore, unique markers in combination with epigenetic modification in the long reads are becoming increasingly useful in deciphering long satellite arrays, such as those in centromeres [32].

### 4.3. Long-Read Assemblies

Long reads, and especially accurate long reads (e.g., those delivered by the consensus HiFi reads from Pacific Biosciences, Menlo Park, CA, USA), are critical not only to improve the assembly quality and contiguity, but also importantly for haplotype phasing (especially in polyploid and allopolyploid genomes). Highly heterozygous genomes, high repeat content, and the presence of segmental duplications all add to the challenge. Several recent algorithms aim to address it. Segmental Duplication Assembler (SDA) [101] enables the partition of the assembly into distinct paralogs, recovering copy-number-variable paralogs that are absent from the human reference genome. To aid phasing, one can make use of the parental genomes (via “trio binning” [102] and derived algorithms). In this approach, each part of an assembly is partitioned into haplotypes (pre-binning strategy) and each haplotype is assembled separately with corresponding reads. This is implemented in HiCanu [91], a modification of the Canu assembler for HiFi reads. In contrast, hifiasm uses graph-binning strategy, allowing the correction of misassigned reads, and attempting to resolve all haplotypes, thus consistently delivering larger assembly contiguity [29]. Ultra-long Oxford Nanopore reads are structurally accurate and can be used to anchor highly accurate assembled HiFi contigs. This strategy was employed to produce a complete assembly of the human chromosome 8 by the T2T consortium [31]. Other strategies require no pedigree information for phasing and combine long reads with Hi-C [103] or single-cell strand sequencing data [104], or make use of several sequencing technologies [105]. Importantly, even if the genome size remains unaffected by the choice of an assembler or assembly parameters, the gene assembly can still be affected, especially when assembling highly heterozygous genomes [106]. This is due to regional sequence expansions or collapses in difficult-to-assemble regions [106]. To conclude, perfect haplotype-resolved assemblies with accurate MHC variants, satellite DNAs, and segmental duplications, all with complete repeat annotations—are now within reach. Last, I expect that after solving the heterochromatin and satellite repeats within a single individual, the focus will shift towards the problem of repeat positioning within pangenomes.

## 5. Future

A Pan-genome can be defined as a collection of genomic sequences to be analyzed jointly or to be used as a reference [107]. The incorporation of thousands of individuals into a single reference will avoid “reference bias”, and mapping reads to such a pan-genome will improve variant calling, especially in regions with a high density of complex variants [107]. While many of the proposed pan-genome implementations represent genomes as graphs with shared and private variants, some of the new approaches have proposed elegant ways of creating pan-genome graphs while preserving linear coordinates [108]. In the future, ultra-long accurate reads, coupled with complete reference pan-genomes, will enable the full understanding of the underlying functional variation hidden in the repetitive parts of the genome. Until then, the considerations outlined in this review, such as reference choice, repeat masking, proper representation of sex chromosomes, and appropriately dealing with multi-mapping reads, will remain essential.

## Figures and Tables

**Table 1:** The comparison in a proportion of mapped reads (%) when using the whole-genome reference, compared to individual chromosomes. Both full and repeat-masked references are contrasted. The SRR622461 dataset of the NA12878 female individual was mapped with bwa mem version 0.7.17-r1188 and default parameters to either unmasked or masked human reference genome hg38. All reads were mapped either to the full reference or to the respective chromosome only.

**Chromosome Name**	**1**	**2**	**3**	**4**	**5**	**6**	**7**	**8**	**9**	**10**	**11**	**12**
mapping proportion [%] (to hg38)	7.6	7.7	6.6	6.4	6.0	5.4	5.0	4.6	3.9	4.3	4.2	4.2
mapping proportion [%] (to itself)	33.8	34.7	32.1	32.4	31.5	30.6	31.5	30.0	30.3	30.7	29.0	29.3
mapping proportion [%] (to masked hg38)	5.2	5.3	4.4	4.1	3.9	3.8	3.4	3.2	2.6	3.0	3.3	2.8
mapping proportion [%] (to masked itself)	6.2	6.5	4.0	4.0	3.7	3.6	4.5	3.0	3.6	4.5	3.1	2.7
**Chromosome Name**	**13**	**14**	**15**	**16**	**17**	**18**	**19**	**20**	**21**	**22**	**X**	**Y ^1^**
mapping proportion [%] (to hg38)	3.2	2.8	2.5	2.7	2.4	2.5	1.6	2.1	1.4	1.2	4.9	0.2
mapping proportion [%] (to itself)	27.7	28.6	27.3	27.7	27.6	26.9	25.0	26.9	25.8	26.0	29.9	23.6
mapping proportion [%] (to masked hg38)	2.2	2.0	1.8	2.0	1.7	1.7	0.9	1.6	0.9	0.8	2.7	0.4
mapping proportion [%] (to masked itself)	2.1	2.0	1.8	3.1	3.1	1.7	1.0	2.7	1.2	2.8	2.6	2.1

^1^ Note the spurious hits to the Y chromosome using the female reads.

## Data Availability

The data in Table 1 were generated with the use of the NA12878 individual from the 1000 Genomes Project Consortium [109] and SRR622461 run. Masked and unmasked reference genome hg38 produced by the Genome Reference Consortium was downloaded from the UCSC website.
